# Inconsistencies in the Assessment of Endodontic Outcomes in Patients with Special Health Care Needs: A Novel Proposal

**DOI:** 10.3390/diagnostics16101426

**Published:** 2026-05-07

**Authors:** Pedro Diz Dios, Alfonso Souto Míguez, Lucía García-Caballero, Eliane García Mato, Márcio Diniz-Freitas, Berta Rivas Mundiña

**Affiliations:** Medical-Surgical Dentistry Research Group, Health Research Institute of Santiago de Compostela, School of Medicine and Dentistry, Santiago de Compostela University, 15703 Santiago de Compostela, Spain; alfonso.souto@gmail.com (A.S.M.); lucia.garcia.caballero@usc.es (L.G.-C.); marcio.diniz@usc.es (M.D.-F.); berta.rivas@usc.es (B.R.M.)

**Keywords:** endodontics, root canal treatment, outcome criteria, disability, special care dentistry

## Abstract

**Conventional**: Endodontic outcome criteria established by the American Association of Endodontists (AAE) and the European Society of Endodontology (ESE) rely heavily on radiographic, clinical, and functional parameters. These criteria may not be applicable to patients with special health care needs, who often present with limited cooperation, communication impairments, and altered pain perception. **Objective**: This study aims to propose an adapted classification system for evaluating non-surgical root canal treatment outcomes in this underserved population. **Methods**: Based primarily on the criteria established by the ESE and our clinical experience, a novel classification system was developed, delineating three outcome categories grounded in both clinical and radiographic parameters. This framework deliberately excludes the “functional” criterion and introduces a “not assessable” category. It was retrospectively applied to 217 non-surgical root canal treatments performed in 137 patients with special health care needs, each with a minimum one-year follow-up. Outcomes were categorized as “favorable,” “uncertain,” or “unfavorable.” **Results**: Using the proposed criteria, 87 treatments (40.0%) were classified as “favorable,” 88 (40.5%) as “uncertain,” and 42 (19.3%) as “unfavorable.” By contrast, the application of AAE/ESE standards resulted in a 71.9% “favorable” classification. Most “uncertain” outcomes occurred in patients with neurodevelopmental disorders, where clinical or radiographic evaluation was not feasible. **Conclusions**: We propose adapted clinical and radiographic criteria for assessing non-surgical root canal treatment outcomes in patients with special health care needs, though broader validation is required. The findings suggest that this procedure remains advisable in this population, with fewer than 20% showing an “unfavorable” long-term outcome.

## 1. Introduction

One of the earliest efforts to define standardized outcome criteria in endodontics was made by Strindberg, who, in a landmark study published in 1956, described both clinical and radiographic parameters for treatment success and failure [1]. Since then, multiple alternative classification systems have been proposed. For instance, Byström et al. [2] introduced the terminology “completely healed,” “incompletely healed or healing,” and “not healed.” Friedman and Mor [3] later emphasized that endodontic treatment outcomes should be referenced with respect to healing and disease, introducing for the first time the category of “functional retention”—defined as the maintenance of the tooth in asymptomatic function regardless of radiographic findings. Subsequently, Wu and Wesselink [4] proposed the terms “effective” and “ineffective,” focusing on treatment efficacy, although this classification was later criticized by other authors [5].

In 2005, the American Association of Endodontists (AAE) [6] formally adopted the terms “healed,” “healing,” and “non-healed,” advocating that these descriptors more accurately reflect the patient’s clinical status and disease progression rather than the therapeutic intervention itself. In addition to symptomatic and radiographic assessments of periradicular pathology, the AAE incorporated the criterion “functional”—defined as a treated tooth or root fulfilling its intended role in the dentition. In 2006, the European Society of Endodontology (ESE) [7] published a consensus report delineating the outcome categories of “favorable” and “unfavorable,” based on clinical features (signs, symptoms, and loss of function) and radiographic findings. The report also introduced an “uncertain” category, defined exclusively by the radiographic evolution of periapical lesions.

The unique characteristics of patients with special needs—including limited cooperation, communication difficulties, and/or altered pain perception—poses significant challenges to the assessment of root canal treatment outcomes based on the criteria established by the AAE [6] or the ESE [7]. Some key consensus reports, such as the 2006 ESE guidelines, even list specific conditions, including uncooperative patients, as contraindications for root canal treatment [7]. However, non-surgical root canal treatment has been shown to be feasible and effective in patients with special health care needs [8], with favorable outcomes even when performed under general anesthesia [9,10,11,12]. The objective of this study is to propose an adapted framework for assessing endodontic treatment outcomes in patients with special health care needs and to conduct a pilot exploratory study to test its applicability and impact.

## 2. Materials and Methods

This study was conducted at the Department of Dentistry for Patients with Special Needs at the University of Santiago de Compostela, Spain. Informed consent was obtained from all participants involved in the study or from their legal guardians. Ethical approval was not required in accordance with the institutional regulations of the authors’ university.

Based on the criteria established by the ESE [7], and our clinical experience, we developed a novel classification system to evaluate non-surgical root canal treatment outcomes. The newly proposed classification system categorizes endodontic treatment outcomes into three categories based on clinical and radiographic criteria: “unfavorable,” “uncertain,” and “favorable” (Table 1). The most innovative contributions include the removal of the “loss of function” criterion and the incorporation of the “not assessable” finding—applicable to both radiographic and clinical variables. Each parameter is assigned a specific score, and the final outcome is determined by the cumulative sum of the radiographic and clinical assessments. An “unfavorable” outcome is assigned if either the radiographic or clinical assessment receives a score of 1; an “uncertain” outcome corresponds to a total score of 4 or 5, provided that neither component is scored as 1; and a “favorable” outcome is attributed to endodontic treatments achieving a total combined score of 6 (Table 1).

Finally, a retrospective observational study was conducted, including cases treated in our department over the past 15 years. This study has been written following the STrengthening the Reporting of OBservational studies in Epidemiology (STROBE) guidelines (Figure S1). The inclusion criteria were as follows: individuals with special needs —defined as those for whom oral health interventions may be complicated by physical, sensory, intellectual, mental, medical, emotional, or social impairments, or a combination of these factors [13]; availability of a comprehensive medical and dental history; any preoperative pulpal or periapical diagnosis in permanent dentition requiring non-surgical root canal treatment (regardless of the etiology of pulpal pathology); and a minimum follow-up period of one year post-treatment. The outcomes of the endodontic treatment in the selected cases were assessed by applying the criteria of the AAE [6], those of the ESE [7], and those of the newly proposed classification system.

## 3. Results

A total of 158 clinical records of patients with special health care needs who underwent endodontic procedures were reviewed. After applying the inclusion criteria, 137 patients were selected, 70 females (51.1%) and 67 males (48.9%), with a mean age of 34.2 ± 13.3 years. The age group most represented in the study population was adults aged 25–59 years (74.2%), whereas patients under 15 years (3.7%) and over 60 years (2.8%) were minimally represented. In these patients, a total of 217 endodontic treatments with at least one year of follow-up were performed, of which 122 were carried out in females (56.2%) and 95 in males (43.8%).

The study group consisted predominantly of patients with neurodevelopmental disorders, intellectual disability, and neurological or syndromic conditions (Table 2). The most frequent primary systemic diagnoses were developmental delay (n = 49 patients; 35.8%), cerebral palsy (n = 20 patients; 14.6%), Down syndrome (n = 16 patients; 11.7%), and autism spectrum disorder (n = 14 patients; 10.2%).

A total of 55.8% of the teeth analyzed in this study (n = 121 teeth) had vital pulp at the preoperative stage, whereas 44.2% (n = 96 teeth) were classified as necrotic. Pulpal conditions in all evaluated teeth were a consequence of deep caries (dental trauma cases are managed on an emergency basis and were therefore excluded from the study to avoid operator-related bias).

By applying the criteria of the new classification, the outcome of the endodontic procedures was considered “favorable” in 87 cases (40.0%), compared to 71.9% under the conventional criteria of the AAE and the ESE (Table 3). Forty-two root canal treatments (19.3%) had an “unfavorable” outcome. In the remaining 88 endodontic procedures (40.5%), the outcome was classified as “uncertain.”

These “uncertain” outcomes were observed in 53 patients, the majority of whom (n = 30; 53.3%) had a systemic diagnosis included in Chapter 6 of the International Classification of Diseases (ICD-11) [14], corresponding to “Mental, Behavioral, or Neurodevelopmental Disorders.” The most frequent diagnoses were moderate-to-severe intellectual disability, psychomotor delay, and autism (Table S1). These patients with “uncertain” outcomes attended follow-up visits; however, none allowed for adequate radiographic imaging; in two cases, the physical examination could not be conducted under optimal conditions; and in three others, the patients had a high pain threshold and communication difficulties.

Among endodontic treatments classified as “favorable” (n = 93), 57.0% teeth had vital pulp and 43.0% were necrotic. Among those classified as “unfavorable” (n = 45), the corresponding proportions were 51.1% and 48.9%, respectively. For treatments with an “uncertain” outcome (n = 79), the distribution was 57.0% vital and 43.0% necrotic.

## 4. Discussion

Root canal treatment remains a procedure with limited accessibility for patients with special health care needs, in whom tooth extraction is often favored over conservative therapy, thereby exacerbating the inequality in treatment planning within this population [15]. Factors most commonly cited as barriers to the provision of root canal treatment include limited cooperation, inadequate oral hygiene, and uncontrolled movements [8]. Although it is acknowledged that endodontic treatment in these patients may require specialized management—such as pharmacological behavioral control or performing the procedure in a single visit under general anesthesia—root canal treatment provides a valuable opportunity for tooth retention in this vulnerable group [8].

Endodontic outcomes serve as key indicators for evaluating the effectiveness of treatment in real-world clinical settings, where conditions are frequently suboptimal and cannot be strictly standardized—unlike efficacy, which refers to the performance of an endodontic procedure under ideal and controlled circumstances [16]. The present study proposes a classification system with criteria specifically adapted to assess endodontic treatment effectiveness in patients with special health care needs, derived from the conventional parameters established by the ESE [7].

The biological outcome of root canal treatment in patients with special needs should be assessed through clinical and radiographic examinations following an appropriate period of follow-up [17]. One of the recurring criticisms of existing endodontic outcome classifications is their failure to account for patient-related factors [5]. In this regard, a Core Outcome Set in Endodontics (COS-ENDO) has recently been developed, highlighting the need to prioritize patient-centered outcomes that are often overlooked by healthcare providers [18]. The COS-ENDO for studies of non-surgical root canal treatment includes the following outcomes: tooth survival, pain, signs of infection, radiographic evidence of periradicular healing, success, functional tooth, need for further intervention, and adverse events/complications [19]. However, the applicability of COS-ENDO to patients with special health care needs is likely to be limited, given the specific characteristics of this population.

In the new classification we propose, the criterion of “loss of function”—which is included in both the AAE [6] and the ESE [7] guidelines—has been deliberately excluded. This decision reflects some clinical characteristics commonly observed in our patient population, such as the presence of anterior open bite or exclusive non-oral feeding due to severe swallowing disorders.

Another novel contribution is the incorporation of the “uncertain” category. In conventional guidelines for assessing endodontic outcomes, the term “uncertain” has been used based exclusively on the radiographic evolution of periapical lesions [1,7,20]. In the present proposal, an outcome is defined as “uncertain” primarily when radiographic assessment is not feasible—due to the unavailability of a follow-up radiograph or suboptimal radiographic capture of the apical anatomy—and/or when clinical evaluation is not possible, owing to limitations in performing a proper examination, altered pain thresholds, and/or severe communication difficulties.

It has been reported that the majority of published studies on endodontic treatment outcomes have been assessed using both clinical and radiographic criteria (40%), radiographic criteria alone (30%), or clinical criteria alone (12%) [21]. When applying both the clinical and radiographic criteria of the AAE [6] and the ESE [7], over 70% of the root canal treatments in the present series were classified as “favorable.” Based solely on radiographic criteria for the quality of nonsurgical endodontic treatment, the reported success rate in patients with special health care needs has ranged from 63% to 87% [9,10,11], while estimates in the general population range from 78% to 85% [22]. It has been suggested that most published studies tend to overestimate successful outcomes after root canal treatment, as periapical radiographs are less sensitive than cone beam computed tomography or histological analysis; in addition, dental extractions and retreatments are often not recorded as treatment failures, and recall rates frequently fall below 50% [23]. This represents one of the strengths of the present study, in which follow-up was successfully completed in 89.1% of patients.

This study has several limitations and should be considered only as a preliminary proposal requiring further discussion and validation. Although the methodological framework was based on structured expert consultations, a more consistent approach could be achieved through methods such as the Delphi technique or large-scale consensus. The pilot study is exploratory in nature, and validation in a multicenter setting is essential to avoid single-center biases, confirm clinical utility, and ensure diagnostic accuracy and reproducibility. Future research should include comprehensive statistical validation, including sensitivity, specificity, predictive values, ROC analyses, and inter-/intra-rater reliability tests, to establish the robustness and clinical relevance of the proposed classification system. The analysis of potential outcome-determining factors—whether lesion-related (e.g., presence of periapical pathology), patient-related (e.g., underlying medical condition), or treatment-related (e.g., need for general anesthesia)—falls outside the scope of this study.

## 5. Conclusions

We propose adapted clinical and radiographic criteria for evaluating the outcomes of non-surgical root canal treatment in patients with special health care needs, incorporating the categories “favorable,” “unfavorable,” and “uncertain.” This proposal offers a more realistic perspective on endodontic outcomes and supports the recommendation of this dental procedure in patients with special health care needs, as fewer than 20% present with an “unfavorable” long-term outcome. While acknowledging the exploratory nature of this study, we hope it will stimulate further discussion toward the development of standardized tools for assessing the outcomes of dental procedures in this patient population and contribute to improved clinical decision-making in the future.

## Figures and Tables

**Table 1:** Novel classification system for evaluating non-surgical root canal treatment outcomes, based on radiographic and clinical parameters.

Type of Assessment	Findings	Outcome (Score)
Radiological assessment (at least after 1 year)	Radiographic evidence of a normal periodontal ligament space surrounding the root. Absence or minimal presence of periradicular radiolucency.	Favorable (3)
	A lesion remains unchanged in size or shows evidence of reduction. Not assessable (due to lack of follow-up radiograph or apex not captured in image).	Uncertain (2)
	Development of a new radiolucent lesion following treatment or progression of a pre-existing lesion. Lesion remains unchanged or shows minimal reduction over a 4-year observation period. Evidence of ongoing root resorption.	Unfavorable (1)
Clinical assessment (at least after 1 year)	Absence of clinical signs or symptoms (e.g., pain, swelling, sinus tract). Tooth remains asymptomatic.	Favorable (3)
	Not assessable (insufficient clinical data)	Uncertain (2)
	Presence of clinical signs or symptoms indicative of infection (e.g., pain, swelling, sinus tract). Symptomatic tooth. Extensive coronal destruction, coronal fracture, or structural compromise precluding restoration.	Unfavorable (1)

Overall evaluation criteria: The final outcome is determined by the sum of the radiographic and clinical assessments. Unfavorable: Assigned if either the radiographic or clinical assessment yields an outcome score of 1. Uncertain: Total score of 4 or 5, provided that neither component has been scored as 1. Favorable: Total combined score of 6.

**Table 2:** Distribution of the predominant systemic conditions in the study sample (n = 137).

WHO ICD-11 Classification	Primary Systemic Conditions	n (%)
**Chapter 6:** Mental, behavioral or neurodevelopmental disorders	Developmental delay Autism spectrum disorder (ASD) Others (psychomotor delay, behavioral disorder, bipolar disorder)	49 (35.8%) 14 (10.2%) 9 (6.6%)
**Chapter 8:** Diseases of the nervous system	Cerebral palsy Others (right hemiplegia, leukodystrophy, cerebellar atrophy, degenerative dementia, encephalomyopathy, epilepsy)	20 (14.6%) 10 (7.3%)
**Chapter 20:** Congenital anomalies, deformities and chromosomal abnormalities	Down syndrome Others (Rett syndrome, spina bifida, Dubowitz syndrome, Arnold–Chiari syndrome, Hallermann–Streiff syndrome, Prader–Willi syndrome, Smith–Lemli–Opitz syndrome, Angelman syndrome, Sotos syndrome, Smith–Magenis syndrome, Cri du Chat syndrome, Noonan syndrome)	16 (11.7%) 19 (13.9%)

**Table 3:** Outcome of endodontic treatment in a series of patients with special health care needs, applying conventional criteria and those of the new classification.

	Favorable ^†^ n (%)	Unfavorable ^†^ n (%)	Uncertain ^†^ n (%)
ESE criteria N = 121 *	81 (71.9)	33 (27.2)	1 (0.4%)
AAE criteria N = 121 *	87 (71.9)	34 (28.1)	0
New criteria N = 217	87 (40.0)	42 (19.3)	88 (40.5)

ESE: European Society of Endodontology; AAE: American Association of Endodontists; * 96 endodontic treatments were excluded due to the lack of radiographic control or because the available radiographs were of poor quality. ^†^ These categories correspond to the terms “healed,” “non-healed,” and “healing,” as defined by the American Association of Endodontists.

## Data Availability

The data are available upon request from the authors.

## Supplementary Materials

The following supporting information can be downloaded at: https://www.mdpi.com/article/10.3390/diagnostics16101426/s1, Figure S1. STROBE flowchart outlining the selection of study participants; Table S1. Systemic diagnosis and radiographic and clinical follow-up of patients with an “uncertain” outcome; Table S2. STROBE statement—checklist of items that should be included in reports of cohort studies.

## Abbreviations

The following abbreviations are used in this manuscript:

ESE	European Society of Endodontology
AAE	American Association of Endodontists
STROBE	STrengthening the Reporting of OBservational studies in Epidemiology
ICD	International Classification of Diseases
COS-ENDO	Core Outcome Set in Endodontics
