# Supplementary material for: Inconsistencies in the Assessment of Endodontic Outcomes in Patients with Special Health Care Needs: A Novel Proposal
Source: Diagnostics (Basel). 2026 May 7;16(10):1426. doi: 10.3390/diagnostics16101426 (PMC13206158; doi:10.3390/diagnostics16101426)
Supplement: Supplementary file 1 [file diagnostics-16-01426-s001.zip › diagnostics-4224408_Fig.S1.pdf]

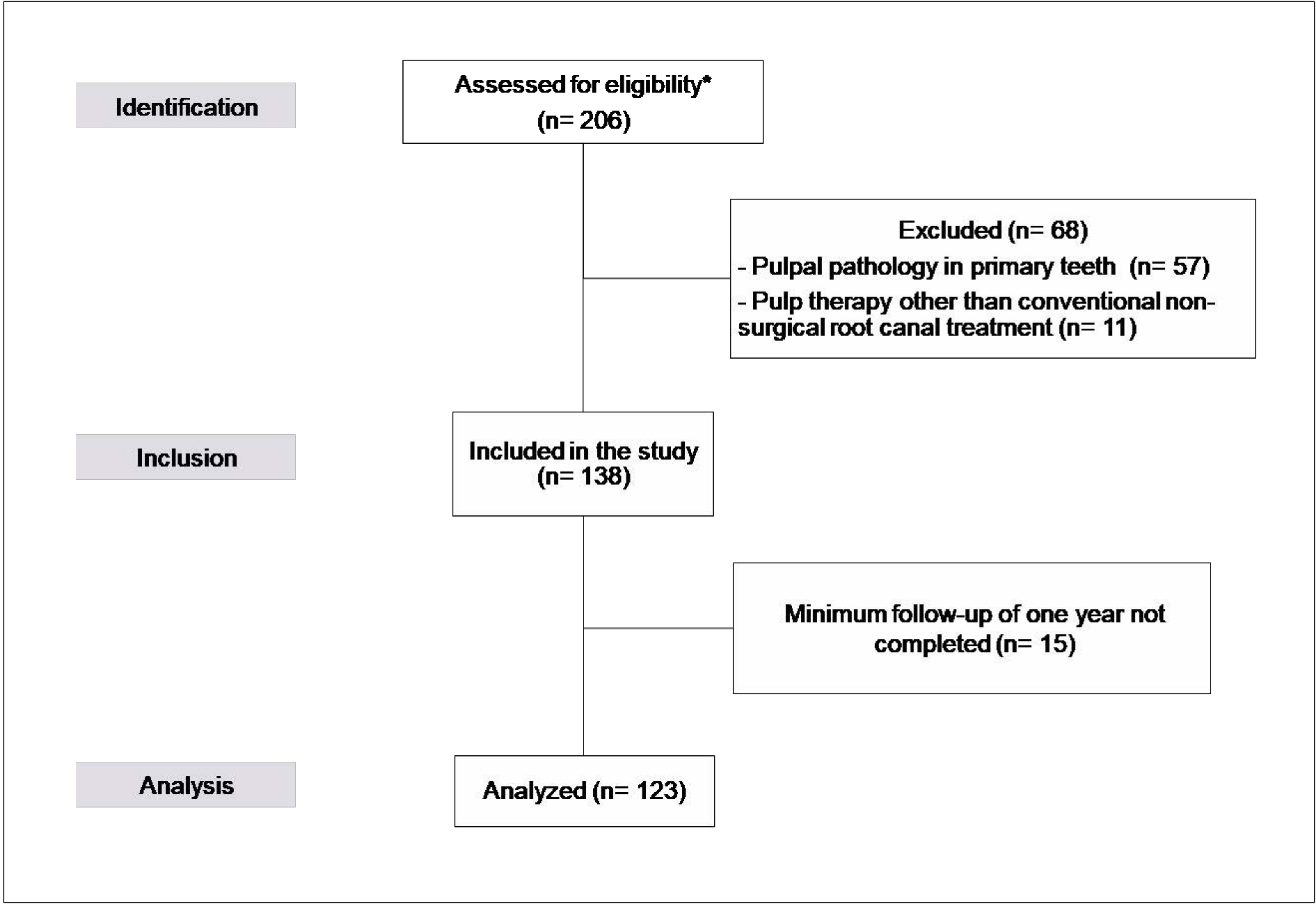
Identification
Assessed for eligibility*
(n= 206)
Excluded (n= 68)
- Pulpal pathology in primary teeth (n= 57)
- Pulp therapy other than conventional non-surgical root canal treatment (n= 11)
Inclusion
Included in the study
(n= 138)
Minimum follow-up of one year not completed (n= 15)
Analysis
Analyzed (n= 123)
