# Supplementary material for: Inconsistencies in the Assessment of Endodontic Outcomes in Patients with Special Health Care Needs: A Novel Proposal
Source: Diagnostics (Basel). 2026 May 7;16(10):1426. doi: 10.3390/diagnostics16101426 (PMC13206158; doi:10.3390/diagnostics16101426)
Supplement: Supplementary file 1 [file diagnostics-16-01426-s001.zip › diagnostics-4224408_Table.S1.pdf]

Table S1. Systemic diagnosis and radiographic and clinical follow-up of patients with an "uncertain" outcome.

Patient	Primary Systemic Diagnosis	ICD-11 Chapter	ICD-11 Disease Code	Follow-up Monitoring		
				Radiographic Findings	Clinical Signs	Clinical Symptoms
1	Cerebral palsy	08	8D20	0		
2	Down syndrome	20	LD40.00	0		
3	Down syndrome	20	LD40.00	0		
4	Psychomotor delay - Epilepsy	06	6A07-8A61	0		
5	Asperger syndrome	06	6A02	0		
6	Intellectual disability	06	6A00	0		
7	Dubowitz syndrome	20	LD27.0Y	0		
8	Down syndrome	20	LD40.00	0		
9	Arnold Chiari malformation - Epilepsy	20	LA07.4-8A62	0		
10	Cerebral palsy	08	8D20	0		
11	Cerebral palsy	08	8D20	0		
12	Psychomotor delay	06	6A03	0		
13	Cerebellar atrophy	08	8D20	0		
14	Psychomotor delay	06	6A07	0		
15	Psychomotor delay	06	6A07	0		
16	Down syndrome	20	LD40.00	0		
17	Severe dysthymia	06	6A7Z	0		
18	Down syndrome	20	LD40.00	0		
19	Psychomotor delay	06	6A07	0		
20	Autism - Epilepsy	06	6A02-8A61	0		
21	Epileptic encephalopathy	08	8A62	0		
22	Intellectual disability	06	6A02	0		
23	Intellectual disability - Epileptic encephalopathy	06	6A03-8A62	0		0
24	Intellectual disability	06	6A02	0		
25	Intellectual disability	06	6A00	0		
26	Intellectual disability	06	6A00	0		
27	Down syndrome	20	LD40.00	0		
28	Smith-Lemli-Opitz syndrome	20	5C52.01	0		
29	Epileptic encephalopathy	08	8A62	0		
30	Intellectual disability - Cerebral palsy - Epilepsy	06	6A03-8D20-8A61	0		0
31	Epileptic encephalopathy	08	8A62	0		
32	Intellectual disability	06	6A00	0		
33	Psychomotor delay - Cerebral palsy	08	6A07-8D20	0		
34	Severe behavioral disorder	06	6A05.0	0	0	

35	Psychomotor delay	06	6A07	0		
36	Cerebral palsy	08	8D20	0		
37	Cerebral palsy	08	8D20	0		
38	Angelman syndrome - Autism	20	LD90.0-6A02	0		
39	Intellectual disability	06	6A04	0	0	
40	Cri du Chat syndrome	20	LD41.0	0		
41	Psychomotor delay - Schizophrenia	06	6A07-6A20.2	0		
42	Asperger syndrome	06	6A02	0		
43	Attention-deficit hyperactivity disorder	06	6A05	0		
44	Noonan syndrome	20	LD2F.15	0		
45	Autism - Severe behavioral disorder	06	6A02-6A05.0	0		
46	Psychomotor delay	06	6A07	0		
47	Intellectual disability	06	6A00	0		
48	Intellectual disability - Psychomotor delay	06	6A03-6A07	0		
49	Intellectual disability	06	6A01	0		
50	Smith Lemli Opitz syndrome	2	5C52.01	0		
51	Autism - Severe behavioral disorder - Intellectual disability	06	6A02-6A05.0-6A02	0		0
52	Autism	06	6A02	0		
53	Intellectual disability	06	6A01	0		

ICD-11: International Classification of Diseases, 11th Revision (ICD-11), developed by the World Health Organization (WHO). ICD-11 Chapter 08: Diseases of the Nervous System. ICD-11 Chapter 06: Mental, Behavioural or Neurodevelopmental Disorders. ICD-11 Chapter 20: Developmental Anomalies [14].
